# Recent Insights into Therapy Resistance in Osteosarcoma

**DOI:** 10.3390/cancers13010083

**Published:** 2020-12-30

**Authors:** Zachary D. Prudowsky, Jason T. Yustein

**Affiliations:** 1 Texas Children’s Cancer and Hematology Centers and The Faris D. Virani Ewing Sarcoma Center, Houston, TX 77030, USA; yustein@bcm.edu; 2 Department of Pediatrics, Baylor College of Medicine, Houston, TX 77030, USA

**Keywords:** osteosarcoma, chemotherapy resistance, radiation resistance, tumor microenvironment, hypoxia, autophagy

## Abstract

**Simple Summary:**

Osteosarcoma is the most common cancer of the bone for children and adolescents. Although chemotherapy regimens have been widely available and effective for many patients for decades, there have been few reliable options available when these standard regimens fail to adequately cure the disease. This suggests internal resistance mechanisms that allow the tumor cells to survive. Here, we review many of the recent studies that have provided new insights into understanding and overcoming chemo- and radioresistance in osteosarcoma.

**Abstract:**

Osteosarcoma, the most common bone malignancy of childhood, has been a challenge to treat and cure. Standard chemotherapy regimens work well for many patients, but there remain minimal options for patients with progressive or resistant disease, as clinical trials over recent decades have failed to significantly improve survival. A better understanding of therapy resistance is necessary to improve current treatments and design new strategies for future treatment options. In this review, we discuss known mechanisms and recent scientific advancements regarding osteosarcoma and its patterns of resistance against chemotherapy, radiation, and other newly-introduced therapeutics.

## 1. Introduction

Osteosarcoma is the most common malignancy arising from bone, accounting for 2.4% percent of pediatric and adolescent cancers worldwide [1]. Osteosarcoma most typically presents in distal femoral metaphysis, but can also occur in other bones, including the upper extremities, pelvis, or bones of the head and neck [1]. Osteosarcoma tends to spread to the lungs or have “skip lesions” to other bones, and approximately 25% of newly-diagnosed patients present with metastatic disease recognizable by modern radiography [2]. Patients with localized cases are managed with chemotherapy and optimal local control surgery; however, surgeries can often be highly morbid, resulting in endoprosthesis placement or limb amputation, requiring long term physical rehabilitation [3]. Neoadjuvant chemotherapy regimen consisting of methotrexate, doxorubicin (also known as Adriamycin), and cisplatin, which is abbreviated MAP, was first added in the 1970s as a means to reduce the size of the primary tumors, minimize surgical morbidity, and treat distant micrometastases [4]. Since this time, 5-year overall survival (OS) rates for localized cases have approached 70–80%. However, patients with initially metastatic disease, those who relapse after achieving remission, or those who fail to respond to MAP regimens have extremely poor 5-year survival rates at approximately 20% [1,5].

To date, MAP has been the only chemotherapy regimen that has shown to have a significant effect on survival rates for patients with osteosarcoma, despite multiple clinical trials that have attempted to introduce new therapies. The gold standard for the assessment of tumor chemosensitivity is the Huvos grading scale [6], which histopathologically quantifies the amount of tumor necrosis at the time of local control surgery after exposure to neoadjuvant chemotherapy. Tumors with >90% necrosis, or Huvos grade III or IV, are considered to have a favorable prognosis; however, those with <90% are considered to have more intrinsic resistance to chemotherapy and are at more risk of treatment failure [6,7,8]. Recent clinical trials have attempted to intensify adjuvant chemotherapy for poor responding tumors; however, none of these attempts have led to improved survival outcomes [8]. Currently, there are no validated molecular biomarkers at the time of diagnosis that can predict responsiveness to standard therapies. The inability to fully understand and overcome therapeutic resistance has remained one of the most significant roadblocks towards implementing new and effective alternative therapies for patients with osteosarcoma. This review summarizes the known mechanisms of resistance for chemotherapy, radiation, and immunotherapy in osteosarcoma, and highlights recent investigations and future directions in how to circumvent these issues.

## 2. Altered Drug Transport

One of the most prominent and well-characterized mechanisms of multi-drug resistance (MDR) in osteosarcoma is the prevalence of the multidrug resistance 1 gene (*MDR1*), which produces p-glycoprotein (p-gp), or the ATP-binding cassette B1 (ABCB1) and actively pumps drugs such as doxorubicin, cisplatin, and other chemotherapies outside of the cell [9]. Other additional proteins in the ABC protein family also may have roles in active drug efflux, such as ABCA1 and ABCC1. Fanelli et al. used a small molecular inhibitor CBT-1 to prevent ABCB1- and ABCC1-mediated efflux, reversing doxorubicin resistance in vitro [9]. Belsario et al. proposed that an increased ratio of ABCB1/ABCA1 indicates higher doxorubicin resistance [10]. ABCA1 effluxes isopentenyl pyrophosphate, which is known to recruit anti-tumor T-cells, providing a novel immune-stimulatory mechanism to potentially treat osteosarcoma. Their study found that tumors high in ABCB1 expression were often low in ABCA1 expression, thus having simultaneous doxorubicin and T-cell resistance. They were able to reverse this phenotype with nanoparticle-encapsulated zoledronic acid, highlighting a potential mechanism to overcome resistance to doxorubicin and immune escape [10].

The role of p-gp on specific chemotherapy agents remains inconsistent. Liu et al., produced an in vitro CRISPR/Cas9 knockdown of p-gp, resulting in improved doxorubicin sensitivity, without effect on cisplatin sensitivity [11]. Alternatively, He et al. found that shRNA knockdown, or inhibition of p-gp with dofequidar fumarate, induced sensitivity to cisplatin [12]. Roundhill et al. hypothesized that osteosarcoma cancer stem cells (CSCs) have elevated levels of p-gp and a similar transporter, ABCG1 [13]. The cells that survived treatment with doxorubicin had elevated p-gp expression, which correlated with increased resistance to doxorubicin, etoposide, vincristine, and actinomycin D. Additionally, their study found that upregulation of ABCG1 directly correlated to doxorubicin and etoposide resistance [13].

Other drug transport mechanisms are also significant in multi-drug resistant phenotypes in osteosarcoma, as p-gp expression does not show a significant correlation with methotrexate resistance [14]. An important protein that does regulate methotrexate resistance is the reduced folate carrier (RFC), which is responsible for guiding the intracellular influx of the drug into the cancer cell. Wang et al. established a correlation between increased methotrexate resistance and decreased RFC expression, resulting in decreased concentrations of methotrexate isotopes [15]. These findings all suggest that the osteosarcoma cell can survive conventional therapies by preventing intracellular accumulation and thus preventing desired cell death.

## 3. Genomic Factors

The genomics and genetic issues involved in the progression and development of osteosarcoma are complex. While many cancers can be driven by specific mutations and genetic aberrations (for example, *EWS-FLI1* fusions in Ewing sarcoma), no primary driving event has been found in osteosarcoma. Chromothripsis, the genetic anomaly where chromosomes are hyperfragmented and randomly reattached, occurs in a large percentage of osteosarcoma cases, showing that overall genetic disarray is a major characteristic of cancer progression [16]. Several germline mutations can cause osteosarcoma. These are seen in several inherited cancer predisposition syndromes such as *P53* in Li Fraumeni Syndrome, *RB1* in Familial Retinoblastoma, *RECQL4* in Rothmund–Thomson syndrome; and several other rare syndromes [16]. These still represent a small proportion of patients with osteosarcoma, and no inherited mutations give way to druggable targets [16]. Whole genomic sequencing approaches can identify druggable targets in certain subsets of patients, but the overall clinical utility of these diagnostic approaches and the subsequent therapies are still under investigation [17,18,19].

Transcriptomic analyses are emerging as new methods of understanding overall expression patterns and identifying drug-sensitive tumors. Recently, the National Institute of Health created the Therapeutically Applicable Research to Generate Effective Treatments (TARGET) database, which compiles multi-omic data from multiple sources nationally, to inspire new research methods and insights into rare difficult-to-treat pediatric tumors, such as osteosarcoma [20]. Bhuvaneshwar et al. used the TARGET and Inova Pediatric Group Osteosarcoma Patients datasets and identified single nucleotide polymorphisms (SNPs) involved in tumor resistance and low rates of tumor necrosis, including variants of *SLC22A1*, *SLC22A8*, *UGT2B15*, and *CHST12* [21]. Additionally, a study by Niveditha et al. used RNA sequencing analysis on osteosarcoma cells after exposure to cisplatin. They reported how a subpopulation of drug-resistant cells had dysregulations in multiple pathways, such as the PI3K/Akt, MAPK, TGF-β, Ras, and NF-κB pathways [22]. Niveditha et al. published an additional study where they explored transcriptomic patterns of osteosarcoma cells after a “drug holiday” to cisplatin. Cisplatin sensitivity returned after a prolonged break in treatment, however, transcriptomic patterns did not return to their pre-treatment patterns, indicating unique clones that developed in the time after treatment [23]. Yang et al., identified differentially expressed genes (DEGs) in methotrexate-resistant Saos2 cell lines, showing that aminoacyl-tRNA synthesis, p53 bioregulation, and cell cycling genes are specifically dysregulated [24].

Studies such as these bring to light one of the main challenges facing clinicians and researchers involved with osteosarcoma, being that currently there are no clinically-relevant molecular markers that predict response to chemotherapy or overall prognosis. Further multi-omic approaches to pre-clinical investigations may help to identify novel biomarker patterns that could predict or aid in overcoming drug resistance in osteosarcoma.

## 4. Signal Transduction

The canonical Wnt/β-Catenin pathway is implicated in the development of osteosarcoma. This pathway is involved in tumor progression, metastasis, and development of microenvironment factors in osteosarcoma [25]. β-catenin overexpression is associated with an increased risk of metastasis and disease progression, and a recent meta-analysis suggests that overexpression of β-catenin indicates a higher likelihood of lung metastasis and poor prognosis [26]. Increased activation of the β-catenin pathway is also linked to chemoresistance as demonstrated in a study by Nomura et al., which identified that the use of Tegavivint, a small molecular inhibitor of downstream β-catenin targets, was able to overcome chemosensitivity in doxorubicin-resistant patient-derived osteosarcoma cell lines [27]. Schloten et al., also showed that doxorubicin resistance is ameliorated with inhibition of the Wnt/β-catenin pathway with the tankyrase inhibitor, XAV939 [28]. Additionally, Tao et al., also described tripartite motif-containing 37 (TRIM37) as a likely modulator of the Wnt/β-catenin pathway, contributing to the resistance of doxorubicin, cisplatin, and methotrexate, providing another potential target to affect the Wnt/β-catenin pathway and reverse chemoresistance [29].

The Hippo/Yes-associated protein (YAP)/transcriptional co-activator with PDZ-binding motif (TAZ) pathway may play a role in osteosarcoma chemotherapy resistance. When activated, this pathway initiates a cascade of intracellular protein kinases to suppress homologous YAP and TAZ nuclear translocation. In absence of this activation, YAP and TAZ would induce transcription of genes that are emerging as drivers of osteosarcoma progression [30,31]. Wang et al. showed that treating MG63 and U2OS cells with doxorubicin and methotrexate induced mammalian sterile 20-like kinase 1 (MST1) protein degradation, thus preventing YAP phosphorylation, implicating this pathway in resistance to these two drugs [32].

Aberrant activation of the Sonic Hedgehog (SHH) pathway is another signal transduction pathway that has implications in osteosarcoma development, progression, and metastasis [33]. A study by Shu et al. implicated a role for this pathway in stemness and chemotherapy resistance and observed that by inhibiting heat shock protein 90 (Hsp90) with the small molecule 17-AAG, they suppressed the SHH pathway and reversed chemoresistance [34].

Additionally, the PI3K/Akt pathway is another potential driver of therapy resistance in osteosarcoma. This pathway has been linked to p-gp expression and therefore chemotherapy resistance. Wei et al. reported that microtubule-affinity regulating kinase 2 (MARK2) overexpression caused upregulation of p-gp through activation of the PI3K/Akt/NF-κB pathway, which enhanced resistance to cisplatin [35]. This study was further validated by Xu et al., who reported that downregulation of MARK2 sensitized CD133+ MG63 and MNNG/HOS cells to cisplatin by PI3K/Akt/NF-κB suppression and modulating DNA protein kinase catalytic subunit effects [36].

**Figure 1:**
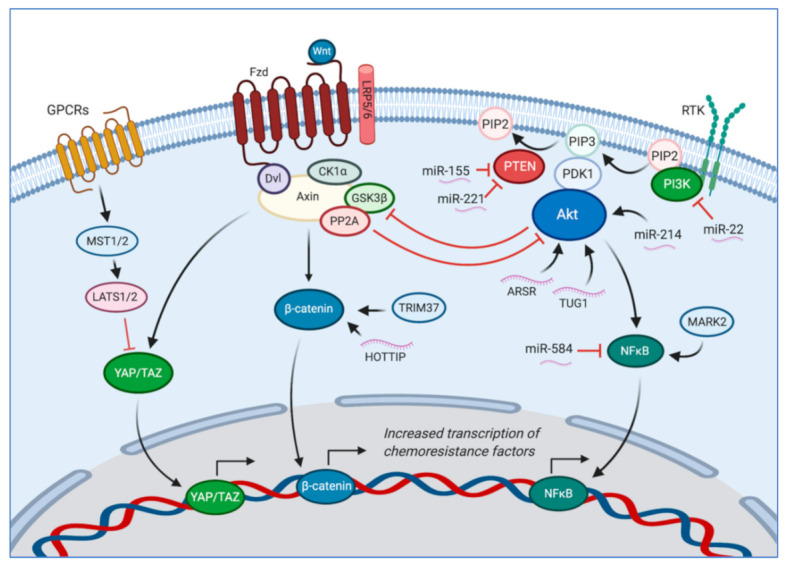
The canonical Wnt/β-catenin pathway, the Hippo/YAP/TAZ pathway, and the PI3K/Akt pathway are each involved and have overlapping features that contribute to chemotherapy resistance and overall osteosarcoma progression. In the absence of Wnt, β-catenin and YAP/TAZ are bound to the Axin protein complex. YAP/TAZ recruits additional enzymes that ubiquitinate β-catenin and cause its degradation. Wnt activates the Frizzled (Fzd) and low-density lipoprotein receptor 5/6 (LRP5/6) coreceptors, which bind to disheveled (Dvl) and the Axin protein complex. This binding causes casein kinase 1α (CK1α) and glycogen synthase kinase 3β (GSK3β) to phosphorylate the cytoplasmic portion of LRP5/6 and further propagate Axin’s release of β-catenin and YAP/TAZ. β-catenin then translocates to the nucleus to activate genes participating in therapy resistance [25]. β-catenin expression is also decreased when lncRNAs HOTTIP is upregulated [37]. TRIM37 also activates the Wnt/β-catenin pathway [29]. Free YAP/TAZ participates in the Hippo pathway, which upon activation causes hyper-phosphorylation of YAP/TAZ and prevents nuclear translocation [30,31]. Additionally, the PI3K/Akt pathway also participates in chemoresistance [35,36]. MiR-22 suppresses PI3K expression [38], while miR-155 and miR-221 suppress PTEN [39,40], miR-214 activates Akt by promoting its phosphorylation [41], and miR-584 inhibits NFκB activity. MARK2 has also been shown to promote activation of the PI3K/Akt/NFκB pathway [35]. ARSR and TUG1 promote Akt upregulation and activation [42,43]. Created with BioRender.com.

An overview of the β-catenin, Hippo/YAP/TAZ, and PI3K/Akt pathways, as well as other relevant proteins and noncoding RNA discussed in this review are depicted in Figure 1.

## 5. Autophagy

Autophagy is a catabolic process where autophagosomes break down intracellular organelles, damaged proteins, and other molecular debris to recycle intracellular materials and sustain cellular homeostasis. Under stressful conditions, autophagy can consume intracellular components to provide energy and survival for affected cells [44]. In cancer, autophagy is often used to promote cancer cell survival and sustains chemotherapy and radiotherapy resistance through multiple mechanisms. Autophagy can be a fine balance within a cancer cell–too little autophagy leads to accumulating cytotoxicity, and too much autophagy leads to metabolic depletion and cell death [44,45]. Disrupting this balance has promising implications in novel therapeutic approaches in osteosarcoma, especially by means of overcoming therapy resistance.

In their reviews, Liao et al. and Niu et al. illustrate the molecular mechanisms of autophagy as it applies to osteosarcoma [44,45]. In summary, autophagy is thoroughly regulated at the molecular level, driven by multiple extracellular activators and intracellular signal transduction pathways. Hypoxia, growth factors, and extracellular stressors drive such pathways as the PI3K/Akt/mTOR pathway, Ras/Raf, ROS/JNK, and hypoxia-inducible factor-1α (HIF-1α) to activate such proteins as the autophagy-related protein family (ATGs) and the autophagosome-associated protein LC3 to drive the formation of phagophores and autophagosomes. These lysosome derivatives become scavengers for organelles and other damaged components to sustain cellular function [44,45].

Recent studies have shown that aberrant activation of autophagy has implications in resistance to conventional therapeutics, including radiotherapy. Osteosarcoma is known for its significant lack of radiosensitivity. Osteosarcoma rarely ever responds to radiation, thus reserving its use to palliative or heroic settings. Feng et al. proposed that hypoxia promoted radioresistance via induction of autophagy [46]. Although the direct roles of hypoxia will be discussed later within this review, it is worth mentioning that autophagy and relative tissue hypoxia have been shown to intimately correlate with one another. Thus, hypoxia-mediated expression of HIF-1α increases LC3 production. The presence of reactive oxygen species (ROS) also correlated with the degree of autophagy activation, likely due to the prevalence of damaged proteins and DNA [46]. Additionally, Jin et al. showed that siRNA knockdown of HIF-1α and treatment with chetomin, a direct inhibitor of HIF-1α-dependent transcription, improved MG63 sensitivity to radiation [47]. Similarly, a study by Ding et al. showed that direct inhibition of autophagy via the molecule 3MA also enhanced radiation-induced cell death of MG63 cells [48]. Oh et al. further characterized the link between autophagy and radioresistance by means of high linear energy transfer (LET) neutron radiation. Their study noted that high LET neutron radiation led to increased autophagy via inhibition of Akt and downstream mTOR activity [49].

Nuclear Factor Epithelioid 2-Related Factor 2 (NRF2) also has a role in promoting autophagy. NRF2 is a transcription factor that helps maintain oxidative stress response and is upregulated in response to radiation. Knockdown of NRF2 reduced autophagy in U2-OS cells and sensitized them to radiotherapy [50].

The estrogen receptor ER*B* may play a role in autophagy regulation as well. This receptor is noted to be upregulated in osteosarcoma cells in vitro and has been known to directly affect the expression of LC3, activating the AMPK pathway, and inhibiting the PI3K/Akt/mTOR pathway. These findings are further discussed in a review by Yang et al. [51]. Overall, these studies provide evidence that targeting autophagy may be necessary to improve response to radiotherapy.

## 6. Noncoding RNAs

New frontiers in cancer research have discovered multiple types of non-coding ribonucleic acids (ncRNA) as modifiers of protein expression and function. NcRNAs were once considered transcriptionally silent, having limited impact on direct protein expression. However, these molecules have been proven to regulate the expression of other genes by anti-sense binding to messenger RNA (mRNA), thus playing a potential role in cancer development [52]. MicroRNA (miRNA) and long-noncoding-RNA (lncRNA) have been investigated as biomarkers [53,54] and implicated in tumorigenesis, metastasis, survival, and other functions in cancer, all of which have been proven true in osteosarcoma, especially regarding therapeutic resistance [52,55].

### 6.1. Micro RNA

MiRNA are small RNA molecules of approximately 20 base pairs in length. MiRNAs are synthesized initially as longer hairpin primary RNAs that are processed and cleaved by the Drosha/DGCR8 complex in the nucleus and the Dicer complex in the cytoplasm. They are then bound to the 3′ untranslated regions of target mRNA via the RISC complex, which keys degradation of the bound mRNA, preventing subsequent translation [52]. MiRNAs are linked to tumor progression, cell cycle regulation, apoptosis resistance, drug resistance, stem cell survival, and metastasis in multiple cancer types [56]. Several studies have investigated the role of circulating miRNA signatures as diagnostic and prognostic biomarkers for multiple cancers [57,58,59,60,61,62,63,64,65,66,67].

Recently, miRNAs have been extensively studied for their role in osteosarcoma. Studies are looking into using circulating miRNA patterns as diagnostic and prognostic biomarkers in osteosarcoma [68,69]. A recent bioinformatic analysis of osteosarcoma miRNA signatures suggests that Bcl-2, VEGFA, CCDN1, PTEN, and Met are central driving factors in osteosarcoma [70].

There have been multiple studies to further investigate individual miRNAs and their role in resistance to chemotherapy and radiation (Table 1). MiRNAs influence the expression of multiple proteins and thus impact any and all involved cellular functions, including signal transduction, autophagy, and many others.

Many miRNAs regulate autophagy and thus result in different chemoresistant phenotypes. For instance, miR-16 and miR-22 both decrease autophagy and cisplatin resistance via downregulation of ATG4B and the PI3K/Akt/mTOR pathway, respectively [38,71]. MiR-101 reduces autophagy by downregulating LC3 and ATG4, promoting doxorubicin sensitivity [72]. Xu et al. presented that miR-140-5p also induces autophagy and chemotherapy resistance to doxorubicin, cisplatin, and methotrexate by downregulation of HMGN5 [73]. MiR-140-5p induces autophagy via IP3k2 and increases resistance to both doxorubicin and cisplatin [74,75].

Two recently-described miRNAs, miR-30a and miR-199a, target Beclin-1 and subsequently suppress autophagy and alter chemoresistance. MiR-30a was found to drive susceptibility to doxorubicin, cisplatin, and methotrexate [73,74], while MiR-199a-5p was found to promote sensitivity to cisplatin [76].

There is conflicting evidence for the role of miR-155 in regulating autophagy and therapy resistance. Chen et al. presented that miR-155 drives doxorubicin resistance by inducing autophagy, although specific targets were not reported [77]; meanwhile, Wang et al., reported that miR-155 drives doxorubicin sensitivity by inducing autophagy and suppressing PTEN expression [39]. Thus, additional studies are required to further elucidate possible context-specific functions for miR-155.

MiRNAs can also influence the expression of proteins involved in drug efflux. For instance, Cheng et al. discovered that miR-137 decreases cisplatin resistance by downregulating ABCB1 and MRP-1 [78]. MiR-153-3p downregulates also ABCB1 and increases cisplatin sensitivity [79]. Additionally, Xie et al. proposed that miR-410-3p downregulates MRP-1 expression by suppressing the NF-κB pathway, decreasing cisplatin resistance [80].

Two additional miRNAs regulate apurinic/apyrimidinic endonuclease 1 (APE1), leading to different resistant phenotypes. MiR-765 increases cisplatin sensitivity by downregulating APE1 [81]–cytoplasmic APE1 has been shown to promote cisplatin and doxorubicin resistance [82], therefore modulation with miR-765 may also drive doxorubicin sensitivity. MiR-513-a-5p suppresses APE1 and has been shown to sensitize osteosarcoma cells to radiation therapy [83]. These suggest varying roles for regulating APE1 in modulating response to treatment.

A comprehensive list of miRNAs that are known to influence therapy resistance, including those discussed above, are listed in Table 1.

**Table 1:** MiRNA that are implicated in osteosarcoma therapy resistance.

Name	Effect on Resistance	Mechanism of Resistance	Citation
MiR-16	Decrease	Sensitizes cells to cisplatin by downregulating ATG4B-driven autophagy; regulated by SNHG16	[71]
MiR-21	Decrease	Reduces cisplatin resistance by targeting Spry2	[84]
MiR-22	Decrease	Enhances cisplatin sensitivity by inhibiting the PI3K/Akt/mTOR pathway and decreasing autophagy	[38]
MiR-29	Decrease	Improves response to methotrexate by suppressing COL3A1 and MCL1 expression	[85]
MiR-30a	Decrease	Suppresses autophagy and promotes doxorubicin-, cisplatin-, and methotrexate-induced cell death by inhibiting Beclin-1	[73,74]
MiR-101	Decrease	Prevents autophagy via suppression of LC3 and ATG4 and improves doxorubicin resistance	[72]
MiR-130a-3p	Decrease	Enhances cisplatin sensitivity via modulating SP1 expression; regulated by lncRNA MIR17HG	[86]
MiR-134-5p	Decrease	Enhances cisplatin sensitivity by targeting MBTD1; regulated by lncRNA TTN-AS1	[87]
MiR-137	Decrease	Increases sensitivity to cisplatin by promoting the expression of MRP-1, GSTp, and ABCB1; regulated by lncRNA NCK-AS1	[78]
MiR-137-3p	Decrease	Decreases doxorubicin resistance by suppressing PTN	[88]
MiR-143	Decrease	With lnc-SARCC, promotes sensitivity to cisplatin via Warburg effect by targeting Hexokinase 2	[79]
MiR-153-3p	Decrease	Increases cisplatin sensitivity by downregulating ABCB1; regulated by lncRNA ROR	[89]
MiR-155	Decrease	Suppresses PTEN expression and downstream autophagy, enhancing doxorubicin sensitivity	[39,74]
MiR-187	Decrease	Enhances sensitivity to doxorubicin by suppressing MAPK7	[90]
MiR-192	Decrease	Reduces methotrexate resistance by suppressing the expression of MMP9, cMyc, KRas, CXCR4, and ADAMTS	[91]
MiR-199a	Decrease	Enhances cisplatin sensitivity via inhibiting HIF-1a	[92]
MiR-199a-5p	Decrease	Inhibits autophagy and enhances cisplatin chemosensitivity by inhibiting Beclin-1	[74,76]
MiR-200b-3p	Decrease	Increases doxorubicin sensitivity by suppressing fibronectin 1 expression; regulated by OIP5-AS1	[93]
MiR-320a	Decrease	Improves sensitivity to doxorubicin via inhibition of Mcl-1; regulated by SNHG12	[94]
MiR-375	Decrease	Increases sensitivity to cisplatin via repression of Mcl-1	[95]
MiR-377-3p	Decrease	Increases sensitivity to cisplatin by suppressing FOSL2; regulated by OIP5-AS1	[96]
MiR-410-3p	Decrease	Sensitizes osteosarcoma to cisplatin by suppressing cyclin D1 and MRP-1; regulated by lncRNA NORAD	[80]
MiR-424-5p	Decrease	Downregulates TFAP2C expression and decreases doxorubicin resistance; regulated by lnc00922	[97]
MiR-499a	Decrease	Promotes sensitivity to erlotinib via suppressing SHKBP1	[98]
MiR-509-3p	Decrease	Sensitizes cells to cisplatin by direct downregulation of AXL and indirect downregulation of ATM	[99]
MiR-513a-5p	Decrease	Promotes sensitivity to radiotherapy by inhibiting APE1	[74,83]
MiR-584	Decrease	Promotes cisplatin and taxane sensitivity by targeting CCN2 and interfering with the NFκB pathway	[100]
MiR-765	Decrease	Downregulates APE1, therefore, increasing sensitivity to cisplatin	[81]
MiR-140-5p	Increase	Promotes doxorubicin and cisplatin resistance through targeting IP3k2 and inducing autophagy	[74,75]
MiR-140-5p	Increase	Increases resistance to doxorubicin, cisplatin, and methotrexate by downregulating HMGN5 and increasing autophagy	[74,101]
MiR-155	Increase	Induces doxorubicin and cisplatin resistance by increasing autophagy	[74,77]
MiR-210	Increase	Induces autophagy and doxorubicin resistance; regulated by lncCTA	[102,103]
MiR-214	Increase	Promotes resistance to radiotherapy by downregulating PHLDA2	[41]
MiR-221	Increase	Increases resistance to cisplatin via PTEN and PPP2R2A suppression	[40,104]
MiR-367	Increase	Increases resistance to doxorubicin by inhibiting KLF4	[74,105]
MiR-488	Increase	Activated by hypoxia, regulates Bim and sensitivity to doxorubicin	[106]
MiR-645	Increase	Suppresses IFIT2 expression and increases cisplatin resistance; regulated by LNC00161	[107]

### 6.2. Long Noncoding RNA

LncRNAs are single-strand RNAs of 200 or more base pair sequences that have become a relatively new area in cancer research. They were originally thought of as biologically inactive transcripts of silent genes; however, now they are recognized as transcriptional regulators for multiple cellular functions, including oncogenesis and tumor suppression [102,108]. LncRNAs are studied as prognostic and diagnostic biomarkers, as well as potential therapeutic targets, for multiple cancers [109]. The primary actions of lncRNA are acting as “sponges” for complementary miRNA [52], binding miRNA and suppressing their regulatory functions, allowing mRNA targets to be freely translated. Although scientists are still investigating the roles of lncRNAs in osteosarcoma, several targets have been shown to play an important role in chemotherapy resistance and cancer cell survival (Table 2).

Two lncRNAs are described to modulate the Wnt/β-catenin pathway and influence chemosensitivity in osteosarcoma. The lncRNA HoxA Transcript at the Distal Tip (HOTTIP) activates the Wnt/β-catenin pathway, promoting cisplatin resistance [37].

Taurine upregulated gene 1 (TUG1) and Activated in Renal cell carcinoma with Sunitinib Resistance (ARSR) both modulate the PI3K/Akt pathway to affect chemoresistance. TUG1 was found to drive cisplatin resistance in vitro by upregulating MET and pAkt [43]. TUG1 expression is also increased in doxorubicin-resistant cell lines, and treatment with polydactin suppressed TUG1 expression and suppressed aberrant Akt pathway activity [110]. ARSR also increases doxorubicin resistance by increasing MDR-1 and survivin expression by activating the Akt pathway [42].

Several lncRNAs affect the expression of drug transporting proteins, thus influencing chemoresistance. For instance, Noncoding RNA Activated by DNA Damage (NORAD), Regulator of Reprogramming (ROR), and NCK1-antisense 1 (NCK-AS1) have all been shown to promote cisplatin resistance. NORAD increases MRP-1 expression by suppressing miR-410-3p, leading to increased resistance to cisplatin [80]. ROR also increases cisplatin resistance by sponging miR-153-3p, leading to increased ABCB1 expression [89]. NCK-AS1 suppresses miR-137, leading to upregulation of MRP-1, ABCB1, and GSTp [78]. Lung Cancer Associated Transcript 1 (LUCAT1) also upregulates ABCB1 expression by targeting miR-200c, which has been described to specifically increase methotrexate resistance [111].

Autophagy and resulting chemoresistance are also regulated by lncRNAs. The lncRNA CTA decreases doxorubicin resistance by targeting miR-210 and suppressing autophagy [103]. SNHG16 exhibits its effect on cisplatin resistance by suppressing miR-16, leading to upregulation of ATG4B and increased autophagy [71].

Additional lncRNAs that play roles in promoting therapy resistance are reviewed in Table 2.

**Table 2:** LncRNA is implicated in osteosarcoma treatment resistance.

Name	Effect on Resistance	Mechanism of Resistance	Citation
SARCC	Decrease	With miR-143, promotes cisplatin sensitivity via hexokinase 2 downregulation	[79]
LINC00161	Decrease	Increases cisplatin-mediated necrosis by upregulating IFIT2; regulates miR-645	[102,107,108]
CTA	Decrease	Sensitizes cells to doxorubicin by downregulating autophagy via miR-210	[102,103]
ROR	Increase	Increases ABCB1 expression and resistance to cisplatin by suppressing miR-153-5p	[89]
NCK-AS1	Increase	Increases cisplatin resistance by suppressing miR-137 and upregulating MRP-1, ABCB1, and GSTp	[78]
TTN-AS1	Increase	Increases resistance to cisplatin by sponging miR-134-5p and upregulating MBTD1	[87]
OIP5-AS1	Increase	Sponges miR-200b-3p to suppress fibronectin1 expression and increase doxorubicin resistance; increases cisplatin resistance by targeting miR-377-3p and upregulating FOSL2	[88,93,96,102]
SNHG16	Increase	Increases autophagy and cisplatin resistance by upregulating ATG4B via suppressing miR-16	[71]
MIR17HG	Increase	Increases resistance to cisplatin by suppressing miR-130-3p and SP1 upregulation	[86]
ODRUL	Increase	Increases resistance to doxorubicin by upregulating ABCB1	[102,108,112]
HOTTIP	Increase	Increases resistance to cisplatin by activating the Wnt/B-catenin pathway	[37,102,108]
OMRUL	Increase	Promotes doxorubicin resistance by altering expression ABCB1 and HIF1a	[102,113]
FOXC2-AS1	Increase	Increases expression of ABCB1 via increased FOXC2 expression leads to doxorubicin resistance	[114]
SHNG12	Increase	Associated with doxorubicin resistance via miR-320a suppression and Mcl-1 upregulation	[94]
TUG1	Increase	Activates the MET/Akt pathway to increase cisplatin and doxorubicin resistance	[43,110]

## 7. Tumor Microenvironment

The tumor microenvironment, and the interactions of solid tumors and their surrounding milieu are continuously evolving and perpetuating cancer survival. Osteoblasts, osteoclasts, fibroblasts, mesenchymal stem cells, hematologic progenitor cells, vascular cells, macrophages, and other immune cells, and a complex molecular environment are all within the stroma of bone [115,116]. The microenvironment is a site for multiple communication events, such as angiogenesis, immune stimulation, and signal transduction initiation [117]. Due to genomic instability, osteosarcoma tumors also consist of complex genomic heterogeneity that makes it difficult to target this tumor as a single treatable entity, creating an even more dynamic and heterogeneous microenvironment. These factors influence the tumor’s susceptibility to multiple treatment modalities.

Crenn et al. alluded to the tumor microenvironment as a means of osteosarcoma cell survival by injecting murine MOS-J cells into mice either subcutaneously or intramuscularly, observing that intratibial osteosarcoma tumors were more susceptible to doxorubicin than intramuscular tumors [115]. These findings likely confirm how the bone environment itself protects osteosarcoma cells, allowing for disease progression. The clinical trial OS2006 attempted to limit bone resorption and promote chemotherapy susceptibility by adding the bisphosphonate zoledronate to conventional chemotherapy, although this was stopped early due to futility [118].

The complex interactions between osteosarcoma cells and the local immune system remains a promising target for future therapies. The presence of CD8+ cytotoxic T-lymphocytes (CTLs) in osteosarcoma biopsies correlated with a favorable prognosis, correlating with more aggressive tumors were lacking a significant population of CD8+ T-cells [117,119,120,121]. These findings inspire the use of checkpoint inhibitors, as some osteosarcoma cells are positive for PD-L1, thus turning on CTLs against the tumor [117,119,120]. However, results from initial clinical trials are still limited. The phase 2 PEMBROSARC trial investigated the anti-PD-1 antibody pembrolizumab alternating with cyclophosphamide in a small cohort of patients with osteosarcoma—only 13.3% of patients had a 6-month non-progression [122].

Mesenchymal stem cells (MSCs) within the tumor microenvironment also play roles in promoting chemotherapy resistance. MSCs are nonmalignant connective tissue cells that ultimately communicate with osteosarcoma cells, potentially influencing each other’s dynamic activities [123]. Studies by Avnet et al. showed that osteosarcoma cells co-cultured with MSCs in acidic environments boosted stemness of the osteosarcoma cells via IL-6 and IL-8 secretion, NF-κB activation, and local acidosis: reducing the pH from 7.4 to 6.8 increased resistance to doxorubicin [123]. They suggested that local acidosis activated cytokine release from MSCs and promoted chemoresistance. A similar study by Tu et al. found that MSCs in co-culture with Saos2 and U2-OS cells promoted resistance to doxorubicin and cisplatin, occurring via IL-6-mediated activation of STAT3 on tumor cells and increased expression of MRP1 and p-gp [124]. Additionally, elevated expression of transforming growth factor β (TGF-β) in the tumor microenvironment correlated to low rates of tumor necrosis in a study by Zhou et al., implicating its role in promoting chemoresistance [125]. These studies highlight the roles of MSCs and secreted pro-inflammatory cytokines in strengthening chemoresistance.

Extracellular vesicles are emerging as another promising target in osteosarcoma. Extracellular vesicles are lipid-rich packages of DNA, RNA, proteins, and other small molecules that act as intercellular communication within the network of stromal and cancer cells [126]. These vesicles can share information with other surrounding cells, including immune cells, vascular endothelial cells, and other tumor cells [126]. These have been extensively studied for their role in multi-drug resistance in multiple cancer types [127]. Specific to osteosarcoma, Torreggiani et al. demonstrated that MDR-1 mRNA can be transferred between osteosarcoma cells via extracellular vesicles, further promoting doxorubicin resistance [128]. Mimicking vesicles via micelleplexes and lipid-based nanoparticle complexes to deliver messenger molecules such as mRNA or miRNA to modulate tumor behavior and reverse chemoresistance may be a worthwhile strategy for future therapy design [129,130].

## 8. Hypoxia

A relevant feature of the osteosarcoma microenvironment is its relative tissue hypoxia. Hypoxia, or low oxygen conditions within the tumor microenvironment has been implied as a driving factor in chemotherapy resistance, tumor growth, cell survival, and metastatic potential in a variety of solid tumors, including osteosarcoma. Hypoxia leads to increased anaerobic glycolysis and increased acidity within the tumor microenvironment. Avnet et al. found that decreased extracellular pH prevented the influx of doxorubicin in osteosarcoma cells, even in p-gp negative tumor cells, suggesting that hypoxia indirectly causes doxorubicin resistance [131]. These findings were reversible in vitro with the addition of the proton pump inhibitor omeprazole [131].

Additionally, osteosarcoma cell survival is thought to be largely driven by the induction of hypoxia-inducible factor-1 (HIF-1). HIF-1 has been studied as a potential biomarker in osteosarcoma, as higher expression correlates with worse overall survival (OS) [132,133]. HIF-1 is a heterodimeric transcription factor made of alpha and beta subunits (HIF-1α and HIF-1β) that influences the expression of proteins that promote survival of the cell in an environment of low-oxidation potential. Here, we discuss its phenotypic effects on resistance to chemotherapy.

HIF-1-dependent and -independent mechanisms of chemotherapy resistance have been proposed in osteosarcoma. Roncuzzi et al. showed that p-glycoprotein expression is induced via HIF-1α in hypoxic conditions, directly causing doxorubicin efflux in MG63 cells. They also noted c-Myc downregulation and p21 upregulation in both doxorubicin-resistant MG63 clones as well as those expressing high levels of HIF-1a, which was thought to promote osteosarcoma cell survival [134].

However, Adamski et al., showed that knockdown and inhibition of HIF-1α did not adequately reverse resistance to doxorubicin, cisplatin, and etoposide in hypoxic condition for HOS, U2-OS, and 791T cells, alluding to HIF-1α-independent mechanisms of chemotherapy resistance [135]. Zhao et al., revealed a synergistic effect of gamboic acid (a caspase-8 activator) and cisplatin in hypoxic conditions in vitro that was maintained despite direct molecular inhibition of HIF-1α, further supporting HIF-1α-independent mechanisms of chemotherapy resistance in hypoxic conditions [136].

Hypoxia modulates other signal transduction pathways. For instance, hypoxia downregulates Wnt signaling via HIF-1α-dependent and -independent mechanisms to suppress the response to doxorubicin. Furthermore, inhibiting the Wnt/β-catenin pathway in hypoxic conditions led to the reversal of doxorubicin resistance [28]. Li et al. showed that hypoxia also increases Notch1 signaling via HIF-1 activation [137]. This subsequently promotes cell cycle progression and multidrug-resistant protein 1 (MRP1) expression, while knockdown of Notch1 significantly downregulated MRP1 expression and sensitized cells to multiple drugs, even in hypoxic conditions [137]. AMP-activated protein kinase (AMPK) activation in hypoxic conditions also led to doxorubicin resistance [138]. Zheng et al., also reported that hypoxic induction of the nuclear protein Mxd1, a protein that is part of the Myc/Max/Mxd family of proteins, leads to increased cisplatin resistance by regulating PTEN expression and PI3K/Akt activation [139].

Spindle and kinetochore associated complex 1 (SKA1) has a role in hypoxic conditions. Ma et al., used in silico models for hypoxic versus normoxic conditions, and identified that downregulation of SKA1 correlates highly to epirubicin and ifosfamide resistance [140]. In the hypoxic setting, increased HIF-1α leads to downregulation of SKA1, which increases the expression of multiple genes implicated in drug resistance, including ABCB1, ABCC2, and GSTP1 [140]. This is in contrast to a study by Yu et al. where they discovered that SKA1 overexpression decreases expression of folylpoly-y-glutamate synthetase (FPGS), an enzyme that helps to activate methotrexate intracellularly, therefore promoting resistance to methotrexate [141]. This study did not focus on the effect of hypoxia in regards to methotrexate resistance, but these contrasting features at least show the SKA1 may have a dual role in processing different chemotherapeutics.

## 9. Cancer Stem Cells

An emerging concept in osteosarcoma biology acknowledges the idea of cancer stem cells (CSCs), which recognizes a subset of tumor cells within the microenvironment that self-renew and aberrantly mature into osteosarcoma cells. CSCs often coalesce together within a niche microenvironment, promoting nearby environmental factors such as blood vessels and mesenchymal support to ensure prolonged survival [142]. CSCs have high DNA repair mechanisms and are frequently multiplying, driving cancer progression [142]. CSCs also have vast protective mechanisms and are thus thought to be more resistant to chemotherapies than typical cancer cells, thus eliminating these cells are essential to the eradication of disease [142].

CSCs distinctly express surface markers such as CD117 and CD133, and many of the chemoresistance factors discussed in this review, such as MDR-1 and the transport protein ABCG2 are upregulated as well, affecting drug transport and metabolism [143]. For instance, Lee et al. reported that CSC expression of MDR-1 and dihydrofolate reductase (DHFR) significantly predict chemotherapy resistance to doxorubicin and methotrexate, respectively [143]. CSCs express levels of Oct-4 and Nanog, similarly to embryonic stem cells [144].

CSCs drive tumor heterogeneity, which creates subpopulations of osteosarcoma cells that have different survival mechanisms that contribute to therapy resistance, metastasis, and relapse. As Schiavone et al. discuss in their review, having a better understanding and characterization of these CSCs, and having a better knowledge of how tumor heterogeneity impacts therapy resistance and survival, is imperative if we are to improve our current therapies [144].

## 10. Future Directions

Progress in discovering and implementing new effective therapies has been stagnant over the past several decades, largely due again to mechanisms of chemotherapy resistance as discussed throughout this review. To overcome these issues, novel and innovative strategies are essential. Several medicines have been tested in pre-clinical models and may be available for large clinical trials in the future. For instance, the agonist of peroxisome proliferator-activated receptor-gamma (PPAR-γ), pioglitazone, has been shown to reverse cisplatin and doxorubicin resistance by repressing p-gp expression, implicating the combination of pioglitazone with cisplatin and doxorubicin as a potential therapeutic option [145,146]. Tyrosine kinase receptor antagonists (TKIs) are emerging therapeutic options in osteosarcoma, and recent clinical trials have looked into regorafenib as a promising option. Regorafenib is a TKI that inhibits multiple receptors, including vascular endothelial growth factor receptors 1–3 (VEGFR1–3), RET, and KIT, and two phase II studies in adults with metastatic osteosarcoma showed delayed progression and increased survival time for patients who received this medicine [147,148]. In addition, cabozantinib, inhibits VEGFR2 and MET, and 33% of the patients enrolled in the phase II CABONE trial showed non-progression of disease at 6 months [149].

Another promising agent is mifamurtide, a liposomal muramyl dipeptide that binds to toll-like receptors and activates macrophages to initiate immune responses against osteosarcoma. It has been used in Europe in several studies, however, its use in the USA has yet to gain approval from the FDA [150]. Other immune therapies, such as anti-HER2 chimeric antigen receptor T-cells (CAR-Ts), are still novel, and trials are still ongoing.

## 11. Conclusions

Osteosarcoma has proven throughout the years to be a frustrating and difficult malignancy to cure. Although conventional chemotherapy regimens are generally accepted in the oncology world and often have good outcomes, few additional therapies have significantly improved survival rates. Resistance to chemotherapy remains one of the biggest challenges for physicians and scientists to conquer to understand current failures and innovate new treatment strategies. Currently, there are many known mechanisms for treatment resistance: this includes such factors as an altered drug processing, tumor microenvironmental factors, including intratumoral hypoxia, genomic aberrations, dysregulated autophagy, and ncRNA post-transcriptional regulation. With a greater and deeper understanding of these factors, novel insights can translate to clinical breakthroughs, either by improving our current therapies or by creating opportunities for new therapies to come to the bedside.
